# Nursing Affairs in Ireland

**Published:** 1917-12-01

**Authors:** 


### NURSING AFFAIRS IN IRELAND.

The Irish Nursing Board have given notice of their intention to hold a public meeting in Dublin for nurses at an early date not yet announced. This is another page taken from the programme of the College of Nursing. But I regard all public meetings of this kind as excellent, no matter under whose auspices they are held. As I have frequently pointed out, the rank and file of the nurses in this country were inarticulate until the College unfurled its flag, and they consequently suffer not only from pardonable but unavoidable ignorance, as well as from that suspicion which isolation engenders. The atmosphere of a public meeting is the best possible cure, and I suggest that every such opportunity ought to be taken by the College Board to spread the light. The three organisations now endeavouring to find recruits in the profession are in actual fact all out to accomplish the same thing or thereabouts, State Registration being the chief objective, and the organisation and betterment of the nurse coming next. The Irish Nursing Board and the Irish League of Trained Nurses differ from the College mainly in their desire to stand aloof from their sisters in the profession outside Ireland, an attitude that obviously and necessarily makes for retrogression. I doubt very much if this principle will find general approval when it is properly understood and its effects realised. The greatest keenness has been shown by midwives in their endeavour to have their Bill so framed as to make them eligible for employment in Great Britain, and the same will apply to every branch of the profession. Hence the necessity for College propagandists to attend all meetings of nurses open to public discussion and set out their aims and those of their opponents with vigour and without reference to the leaders of the opposing camps. It is not the Irish nurses who are at variance, but the organisers of faction and reaction.

### The Midwives Bill.

Speaking generally, this Bill has been received with a chorus of approval, and the hope is expressed that its passage through Parliament may be speedy. Certain reasonable amendments, however, are suggested in order to render the Act effective. One of these is to provide for a larger number of midwives on the Central Board, the Bill providing for only one. Seven are suggested, one for each of the four provinces and one each for the cities of Dublin, Belfast, and Cork. Of course, the expert midwife is the doctor, and the Bill provides for eight of these serving on the Board. Nevertheless, this is distinctly a branch of women's work, and, moreover, this is woman's hour, and while seven appears to be an extravagant demand, the suggestion of one midwife only would appear to be an error on the other side. A second suggestion is that there should be some provision for inspection. It is held, for example, that if a woman chooses to open a private maternity home, even if she is a certified midwife, her establishment ought to be open to inspection. This, I have no doubt, is contemplated by the authors of the measure, but probably rather for the local authority than for the Central Board. There is a great deal to be said in favour of independent headquarters inspection, as the undercurrents of local communities are calculated to hinder effectual supervision, especially in the less populous districts, where personal influence and petty wirepulling count for much. I understand that a certain amount of opposition to the Bill exists in medical circles, as it is doubtless felt that the entrance of the certified midwife on the scene may cause a diminution of fees. This is a purely personal grievance, and must be stoutly withstood. The cause of child-welfare was never so urgently in need of championing, and there is no room for doubt that the nefarious practice of the handy woman acts as a scourge in poor neighbourhoods. The Bill as it stands is excellent, and every effort must be made to secure its passage into law. If it can be suitably amended so as to make its operations more effective, all the better.

IERNE.

